# Prognostic Nutritional Index is a Predictor of Free Flap Failure in Extremity Reconstruction

**DOI:** 10.3390/nu12020562

**Published:** 2020-02-21

**Authors:** Jihion Yu, Joon Pio Hong, Hyunsuk Peter Suh, Jun-Young Park, Doo-Hwan Kim, Seungsoo Ha, Joonho Lee, Jai-Hyun Hwang, Young-Kug Kim

**Affiliations:** 1 Department of Anesthesiology and Pain Medicine, Asan Medical Center, University of Ulsan College of Medicine, 88, Olympic-ro 43-gil, Songpa-gu, Seoul 05505, Korea; yujihion@gmail.com (J.Y.); parkjy@amc.seoul.kr (J.-Y.P.); knaaddict@gmail.com (D.-H.K.); hac541309@gmail.com (S.H.); tishiya@naver.com (J.L.); jhhwang11@hotmail.com (J.-H.H.); 2 Department of Plastic Surgery, Asan Medical Center, University of Ulsan College of Medicine, 88, Olympic-ro 43-gil, Songpa-gu, Seoul 05505, Korea; joonphong@amc.seoul.kr (J.P.H.); hyunsuk.suh@amc.seoul.kr (H.P.S.)

**Keywords:** prognostic nutritional index, free flap failure, extremity reconstruction

## Abstract

The nutritional condition of patients is an important prognostic factor in various diseases. Free flap failure is a serious complication in patients undergoing free flap reconstruction, increasing morbidity and hospital costs. We evaluated the predictive factors, including the prognostic nutritional index (PNI), associated with free flap failure in extremity reconstruction. The PNI was calculated as follows: 10 × serum albumin (g/dL) + 0.005 × total lymphocyte count (per mm^3^), with a PNI <40 defined as low. Univariate and multivariate logistic regression analyses were performed to evaluate factors predictive of free flap failure. Postoperative outcomes, including duration of hospital stay and rate and duration of intensive care unit admission, were also evaluated. Of the 625 patients included, 38 (6.1%) experienced free flap failure. Multivariate logistic regression analysis revealed that predictors of free flap failure were female (odds ratio: 2.094; *p* = 0.031) and a low PNI (odds ratio: 3.859; *p* <0.001). The duration of hospital stay was significantly longer in patients who did than those who did not experience free flap failure (62.1 ± 55.5 days vs. 28.3 ± 24.4 days, *p* <0.001). A low PNI is associated with free flap failure, leading to prolonged hospital stay. This result suggests that the PNI can be simply and effectively used to predict free flap failure.

## 1. Introduction

Microvascular free flap reconstruction is considered an optimal treatment option in patients with soft tissue defects [1,2]. Free flap failure, however, can occur, even when reconstruction is performed by experienced surgeons. Various patient and surgical factors have been associated with free flap failure, including age, diabetes mellitus, smoking history, and operation time [3,4,5,6]. Free flap failure results in additional surgery, prolonged hospital stay, and increased hospital costs [7]. Therefore, it is important to identify factors associated with free flap failure in patients undergoing microvascular free flap reconstruction.

Malnutrition has been found to have negative effects on the immune system and inflammatory responses, impairing the wound healing process [8]. The prognostic nutritional index (PNI) has been widely used to evaluate the nutritional status of patients [9]. The PNI, which is calculated based on serum albumin concentration and blood lymphocyte count, is a useful indicator of postoperative complications, systemic inflammation, and overall survival in cancer patients. Previous studies evaluating risk factors for free flap failure in patients who underwent free flap reconstruction did not assess whether the PNI, an indicator of nutritional and immunologic status, was predictive of free flap failure. To our knowledge, only one study to date has evaluated the effect of serum prealbumin concentration, an indicator of acute malnutrition, on free flap failure [10].

This study was designed to evaluate predictive factors, including the PNI, related to free flap failure in patients who underwent free flap reconstruction of the upper and lower extremities. In addition, postoperative outcomes, such as length of hospital stay and rate and length of intensive care unit admission, were compared in patients who did and did not experience free flap failure.

## 2. Methods

### 2.1. Patients

Patients who underwent free flap reconstruction of the upper and lower extremities at the Asan Medical Center, a tertiary medical center in Seoul, Korea, between January 2010 and December 2017, were retrospectively recruited. Patients aged < 19 years old, patients with incomplete medical records, and patients who underwent free flap combined with another surgery were excluded. Medical records were reviewed and free flap failure was noted. This study was approved by the institutional review board of the Asan Medical Center (approval no. 2019–1025).

### 2.2. Anesthesia Protocol

Beginning at induction of anesthesia, the following were monitored in all patients: electrocardiography, pulse oximetry, noninvasive blood pressure, end-tidal carbon dioxide concentration, and bispectral index (Aspect Medical Systems, Inc., Newton, MA, USA). General anesthesia was induced with 1.5–2 mg/kg propofol or 4–5 mg/kg thiopental sodium and 0.6 mg/kg rocuronium, and was maintained with 1.5–2.5 vol% sevoflurane with 50% nitrous oxide and 50% oxygen. Bispectral index was maintained at 40–60 by adjusting the concentration of sevoflurane, and arterial pressure was monitored continuously by arterial catheterization. Crystalloid fluid with lactated Ringer’s solution or plasma solution A (CJ Pharmaceutical, Seoul, Korea) and synthetic colloid fluid with 6% hydroxyethyl starch were administered. The crystalloid fluid was administered at a rate of 4–6 mL/kg/h, and the synthetic colloid fluid was administered when an estimated blood loss of >500 mL occurred during the free flap surgery. Red blood cells (RBCs) were transfused when hemoglobin concentration was below 8 g/dL. Patients were administered vasopressors or inotropics, such as ephedrine, phenylephrine, norepinephrine, or dobutamine, when their mean arterial blood pressure was below 60 mmHg for more than 5 min.

### 2.3. Surgical Protocol

Selection of optimal flap type was based on the size and depth of the defect, the length of the pedicle, and patient posture during surgery. Flap elevation was performed according to the protocol of our institution [11,12,13]. The flaps used in the present study were anterolateral thigh flap, superficial circumflex iliac artery perforator flap, superior gluteal artery perforator flap, radial free forearm flap, posterior interosseous flap, and gracilis muscle flap. The artery of each harvested flap was anastomosed end to end to the perforator artery or end to side to the peripheral artery, and the vein of each flap was anastomosed end to end. To check the patency of the free flap, the maximal blood flow velocity was measured by duplex ultrasonography (LOGIQ^®^ e; GE Healthcare, Wauwatosa, WI, USA) at the pedicle of the flap, distal to the anastomosis, ten minutes after the arterial and venous anastomoses of the free flap. All operations were performed by two highly experienced surgeons.

### 2.4. Data Collection and Definition

Preoperative variables included age, sex, body mass index, American Society of Anesthesiologist physical status, comorbidities (hypertension, diabetes mellitus, coronary artery disease, heart failure, cerebrovascular accident, dyslipidemia, and chronic kidney disease), smoking history, cause of the defect, defect site, and preoperative laboratory data (hemoglobin, albumin, and creatinine concentrations; white blood cell and lymphocyte counts; and the PNI) examined within 2 weeks before the free flap surgery. Intraoperative variables included duration of anesthesia, duration of surgery, amount of crystalloid administered, amount of colloid administered, RBC transfusion rate, amount of RBCs transfused, use of vasopressor/inotropic, flap perforator type, flap dimension, and flap type. Postoperative outcomes included length of hospital stay, rate of admission to the intensive care unit, and length of stay in the intensive care unit.

Flap failure during hospitalization was defined as partial or complete flap death, indicated by vascular compromise and any degree of necrosis after surgery [14]. Coronary artery disease was defined as a history of angina, myocardial infarction, percutaneous coronary intervention, or coronary artery bypass graft surgery. Heart failure was defined as left ventricular ejection fraction <40%. A cerebrovascular accident was defined as a history of stroke or transient ischemic accident. Dyslipidemia was defined as a history of treatment with any antihyperlipidemic medication or elevated serum lipoprotein concentrations. Chronic kidney disease was defined as a previous diagnosis of chronic kidney disease. Smoking history was classified as three categories: non-smoker, ex-smoker, and current smoker. Causes of defect were classified as diabetes mellitus, trauma, and others, the latter including tumor, chronic osteomyelitis, scar adhesion, and arteriovenous malformation.

The PNI was calculated as 10 × serum albumin (g/dL) + 0.005 × total lymphocyte count (per mm^3^) [9,15], with a PNI <40 defined as low [16,17]. Flap types were classified into four categories: anterolateral thigh flap, superficial circumflex iliac artery perforator flap, superior gluteal artery perforator flap, and others including radial free forearm flap, posterior interosseous flap, and gracilis muscle flap.

### 2.5. Statistical Analysis

Continuous variables were expressed as mean ± standard deviation and categorical variables as number (percent). Continuous variables were compared by Student’s t-test or the Mann–Whitney U-test and categorical variables by the chi-square test or Fisher’s exact test. Independent predictors of free flap failure were identified by univariate and multivariate logistic regression analyses. Variables with a *p*-value <0.05 on univariate logistic regression analyses were included in the multivariate logistic regression analyses. The odds ratios of flap failure based on predictors of free flap failure were calculated by logistic regression analysis. All statistical analyses were performed using SPSS^®^ version 21.0 software (IBM, Armonk, NY, USA), with *p* <0.05 considered statistically significant.

## 3. Results

**Figure 1:**
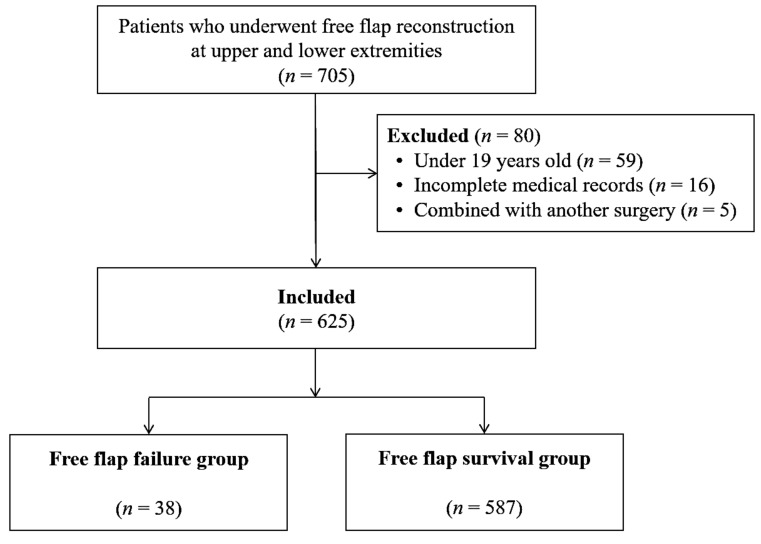
Flowchart of study participants.

A total of 705 patients underwent free flap reconstruction at the upper and lower extremities between January 2010 and December 2017. Eighty patients were excluded, 59 because they were aged <19 years, 16 because their medical records were incomplete, and five because they underwent free flap reconstruction combined with another surgical procedure. Finally, 625 patients were included in the study cohort (Figure 1).

Patient characteristics and intraoperative data are shown in Table 1 and Table 2, respectively. Of the 625 patients, 38 (6.1%) experienced free flap failure. Sex, history of chronic kidney disease, hemoglobin concentration, white blood cell counts, serum albumin concentration, the PNI, and RBC transfusion rate differed significantly between the flap failure and flap survival groups (Table 1 and Table 2).

Multivariate logistic regression analysis revealed that female sex (odds ratio: 2.094; 95% confidence interval: 1.071–4.093; *p* = 0.031) and a low PNI (odds ratio: 3.859; 95% confidence interval: 1.942–7.667; *p* <0.001) were significantly associated with free flap failure in extremity reconstruction (Table 3).

**Figure 2:**
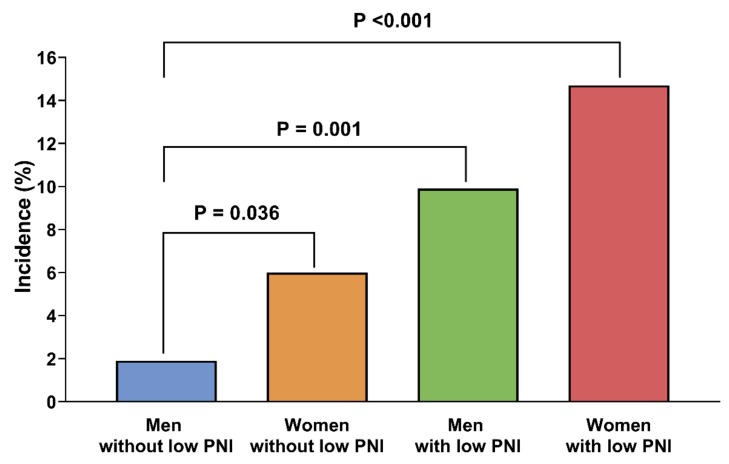
Incidence of free flap failure as a function of the PNI and sex. The PNI was calculated as 10 × serum albumin (g/dL) + 0.005 × total lymphocyte count (per mm^3^), with a PNI <40 defined as low. Abbreviations: PNI, prognostic nutritional index.

When patients were classified according to the PNI and sex, the probabilities of free flap failure were 1.9% (*n* = 5) in men without a low PNI; 6.0% (*n* = 9) in women without a low PNI; 9.9% (*n* = 14) in men with a low PNI; and 14.7% (*n* = 10) in women with a low PNI (Figure 2).

Compared with men without a low PNI, the incidence of free flap failure in extremity reconstruction was significantly higher in women with a low PNI (odds ratio: 8.931; 95% confidence interval: 2.941–27.117; *p <*0.001) (Table 4). The length of hospital stay was significantly longer in the free flap failure than in the free flap survival group (62.1 ± 55.5 days vs. 28.3 ± 24.4 days, *p* <0.001) (Table 5).

## 4. Discussion

The main findings of the present study were that the incidence of free flap failure in 625 patients undergoing free flap reconstruction of the upper and lower extremities was 6.1%, and that a low PNI and female sex were independent predictors associated with free flap failure in this patient cohort. In addition, the duration of hospital stay was significantly longer in the free flap failure than in the free flap survival group. To our knowledge, this study is the first to show that the PNI is significantly associated with free flap failure in extremity reconstruction.

Due to advances in microsurgical techniques, free flap reconstruction has been demonstrated to be a safe and effective surgical treatment for various complex defects [18,19]. However, free flap failure can occur, even when reconstruction is performed by very experienced surgeons. Free flap failure is a serious complication, increasing patient morbidity rates and hospital costs [3]. The incidence of free flap failure in the present study, 6.1%, is consistent with rates reported in previous studies, ranging from 1% to 9% [3,19,20,21].

Malnourished patients are more likely to have a poorer prognosis, including high rates of clinical complications and mortality and increased hospital stay [22]. A complex process is required to assess the full nutritional status of patients, including detailed nutritional intake, symptoms associated with malnutrition or excess, information on body composition, and laboratory tests of nutrition related indicators [23]. The PNI assesses nutrition and inflammation status based on serum albumin concentrations and total lymphocyte counts, parameters that can be easily evaluated in clinical laboratories [15,24]. Albumin is involved in the maintenance of serum osmolality, participates in tissue repair, and regulates systemic inflammation [25]. Hypoalbuminemia results in poor or delayed tissue healing, a reduction in collagen synthesis, and granuloma formation after surgery [26,27,28], as well as increasing the risk of infection by increasing the dead space of the wound. Hypoalbuminemia has also been associated with impaired innate immune responses, altering macrophage activity [29], as well as reducing plasma colloid osmotic pressure, causing tissue edema and leakage of interstitial fluid, which mediates bacterial propagation, into wounds [30,31]. Lymphocyte counts reflect the immune status of patients and the degree of systematic inflammation. Lymphocytes play an important role in controlling immune responses by recruiting and activating transcription factors and inflammatory mediators [32,33]. Therefore, the PNI can affect free flap failure through interactions between nutritional status and systematic inflammatory responses.

The cutoff value for a low PNI corresponding to short-term postoperative complications and mortality has been found to differ among studies. Studies reported cutoff values of 40–51 [34,35,36], with several studies, such as ours, defining a low PNI as below 40 [16,17,37]. Other studies have defined PNIs >50 as normal, PNIs of 45–50 as mild malnutrition, 40–45 as moderate to severe malnutrition, and <40 as serious malnutrition [38,39,40]. Kono et al. reported that a PNI <40, indicative of serious malnutrition, was significantly associated with severe adverse toxicity of radiotherapy among patients with head and neck cancer [40]. Moreover, Onodera et al. demonstrated that patients with a PNI <40 had a high complication rate for operations such as resection and anastomosis in gastrointestinal surgery, and that a PNI <40 was a contraindication for these procedures [15]. In line with previous studies, our study demonstrated that the risk of free flap failure was significantly higher in patients with a PNI <40 than in those with a higher PNI, suggesting the importance of determining nutritional status by the PNI before surgery. Special attention should be paid to patients with a PNI <40 to reduce the risk of free flap failure in extremity reconstruction.

The present study also found that the rate of free flap failure was higher in women than in men. Estrogen can protect vascular endothelial cells from undergoing apoptosis induced by TNF-α [41,42]. In our study, the average age of patients was >50 years, with most women being post-menopausal, thus having low blood estrogen concentrations. In addition, the free flap failure is believed to be affected by vascular properties such as diameter. Women may have smaller vascular diameters than men. Small blood vessels can make vascular anastomosis surgeries more difficult and may be associated with inadequate free flap blood flow perfusion. These factors might partially explain the higher rate of free flap failure in women. Furthermore, the combination of female sex and a low PNI could have synergistic effects, with women having a low PNI being at an approximately 8.9 times higher risk of free flap failure than men without a low PNI.

Although we found that diabetes mellitus was not significantly associated with free flap failure, studies have yielded conflicting results on the association between diabetes mellitus and free flap failure [5,43,44,45]. Several studies found that diabetes mellitus was not significantly associated with free flap failure [43,44], whereas other studies reported associations between diabetes mellitus and free flap failure, surgical site infection, and prolonged hospital stay [5,45]. These conflicting findings may be due to the relatively small sample sizes, differences in defect site and flap type, and definitions of free flap failure.

This study also showed that free flap failure was associated with longer hospital stay, confirming previous results [46,47]. These previous studies reported that hospital stay was longer and hospital costs were higher in patients who did than in those who did not experience free flap failure in breast and head and neck reconstruction. It is therefore important to anticipate and pay special attention to patients with factors predictive of flap failure who are undergoing reconstruction of the extremities.

This study had several limitations. First, it was a retrospective, single-center study, suggesting that our results may not be generalizable to other patient populations. Moreover, we could not evaluate all covariates that may have affected the analysis, although we included all likely covariate factors. Second, this study did not investigate reconstruction at other sites, such as the head and neck and breast; rather, the present study was limited to the extremities. Additional studies are needed to clarify the independent predictors of free flap reconstruction at other sites.

## 5. Conclusions

Free flap failure occurred in 6.1% of patients undergoing free flap reconstruction of the upper and lower extremities. Free flap failure was associated with a low PNI and female sex. The length of hospital stay was longer in patients with than in those without free flap failure. These results suggest that the PNI, an easily measurable parameter, can be simply and effectively used to predict free flap failure in extremity reconstruction. Moreover, perioperative care is especially required in women with a low PNI to reduce the likelihood of free flap failure.

## Figures and Tables

**Table 1:** Patient characteristics.

Variables	All Patients (*n* = 625)	Free flap Failure (*n* = 38)	Free Flap Survival (*n* = 587)	*p-*Value^1^
Age (years)	52.9 ± 15.5	50.1 ± 16.2	53.0 ± 15.5	0.256
Sex (female)	219 (35.0)	19 (50.0)	200 (34.1)	0.046
Body mass index (kg/m^2^)	24.1 ± 3.6	24.0 ± 2.7	24.1 ± 3.7	0.822
ASA physical status				0.242
≤II	534 (85.4)	30 (78.9)	504 (85.9)	
III	91 (14.6)	8 (21.1)	83 (14.1)	
Hypertension	218 (34.9)	15 (39.5)	203 (34.6)	0.540
Diabetes mellitus	211 (33.8)	15 (39.5)	196 (33.4)	0.442
Coronary artery disease	18 (2.9)	0 (0.0)	18 (3.1)	0.273
Heart failure	8 (1.3)	0 (0.0)	8 (1.4)	0.469
Cerebrovascular accident	16 (2.6)	1 (2.6)	15 (2.6)	0.977
Dyslipidemia	107 (17.1)	9 (23.7)	98 (16.7)	0.268
Chronic kidney disease	94 (15.0)	10 (26.3)	84 (14.3)	0.045
Smoking history				0.281
Non-smoker	137 (21.9)	11 (28.9)	126 (21.5)	
Ex-smoker	161 (25.8)	6 (15.8)	155 (26.4)	
Current-smoker	327 (52.3)	21 (55.3)	306 (52.1)	
Cause of defect				0.223
Diabetes mellitus	174 (27.8)	15 (39.5)	159 (27.1)	
Trauma	190 (30.4)	11 (28.9)	179 (30.5)	
Others^2^	161 (41.8)	12 (31.6)	249 (42.4)	
Defect site				0.445
Upper extremities	54 (8.6)	2 (5.3)	52 (8.9)	
Lower extremities	571 (91.4)	36 (94.7)	535 (91.1)	
Preoperative laboratory tests				
Hemoglobin (g/dL)	12.0 ± 2.2	10.8 ± 1.8	12.1 ± 2.2	<0.001
White blood cells (/mm^3^)	7100.6 ± 2741.1	8026.3 ± 3293.6	7041.7 ± 2693.8	0.033
Lymphocytes (/mm^3^)	1917.1 ± 734.3	2004.0 ± 729.2	1921.4 ± 735.5	0.502
Albumin (g/dL)	3.5 ± 0.8	3.0 ± 0.8	3.5 ± 0.8	0.001
Prognostic nutritional index^3^	43.8 ± 9.1	38.6 ± 8.8	44.1 ± 9.1	<0.001
Prognostic nutritional index (<40)	210 (33.6)	24 (63.2)	186 (31.7)	<0.001
Creatinine (mg/dL)	1.4 ± 1.6	1.5 ± 1.8	1.3 ± 1.5	0.468

Continuous variables are presented as mean ± standard deviation and categorical variables as number (percent). ^1^For comparisons between the flap failure and flap survival groups. ^2^Tumors, chronic osteomyelitis, scar adhesion, and arteriovenous malformation. ^3^Calculated as follows: 10 × serum albumin (g/dL) + 0.005 × total lymphocyte count (per mm^3^). Abbreviation: ASA, American Society of Anesthesiologists.

**Table 2:** Intraoperative data.

Variables	All Patients (*n* = 625)	Free Flap Failure (*n* = 38)	Free flap Survival (*n* = 587)	*p*-Value^1^
Duration of anesthesia (min)	382.8 ± 127.5	410.9 ± 135.3	381.0 ± 126.9	0.162
Duration of operation (min)	267.9 ± 124.7	293.1 ± 118.8	266.2 ± 125.0	0.198
Crystalloid administered (mL)	2489.5 ± 1141.4	2813.7 ± 1461.7	2468.6 ± 1115.8	0.071
Colloid administered (mL)	207.9 ± 474.00	213.2 ± 305.1	207.5 ± 483.1	0.943
RBC transfusion rate	118 (18.9)	12 (31.6)	106 (18.1)	0.039
Amount of RBCs transfused (unit)	0.5 ± 1.5	0.9 ± 1.4	0.5 ± 1.5	0.177
Vasopressor/inotropic	402 (64.3)	29 (76.3)	373 (63.5)	0.111
Flap perforator				0.134
Skin	82 (13.1)	1 (2.6)	81 (13.8)	
Fasciocutaneous	37 (5.9)	3 (7.9)	34 (5.8)	
Musculocutaneous	506 (81.0)	34 (89.5)	472 (80.4)	
Flap dimension (cm^2^)	129.2 ± 170.8	168.0 ± 158.5	126.7 ± 171.4	0.148
Flap type				0.220
ALT	228 (36.5)	17 (44.7)	211 (35.9)	
SCIP	316 (50.6)	17 (44.7)	299 (50.9)	
ALT + SCIP	13 (2.1)	2 (5.3)	11 (1.9)	
SGAP	27 (4.3)	2 (5.3)	25 (4.3)	
Others^2^	41 (6.6)	0 (0.0)	41 (7.0)	

Continuous variables are presented as mean ± standard deviation and categorical variables as number (percent). ^1^For comparisons between the flap failure and flap survival groups. ^2^Radial free forearm flap, posterior interosseous flap, and gracilis muscle flap. Abbreviations: RBC, red blood cell; ALT, anterolateral thigh; SCIP, superficial circumflex iliac artery perforator; SGAP, superior gluteal artery perforator.

**Table 3:** Univariate and multivariate logistic regression analyses of factors predictive of free flap failure.

Variables	Univariate Analysis		Multivariate Analysis	
	Odds Ratio (95% CI)	*p*-Value	Odds Ratio (95% CI)	*p*-Value
Age	0.988 (0.968–1.009)	0.257		
Sex (female)	1.935 (1.002–3.738)	0.049	2.094 (1.071–4.093)	0.031
Body mass index	0.989 (0.902–1.085)	0.822		
ASA physical status	1.619 (0.718–3.654)	0.246		
Chronic kidney disease	2.139 (1.002–4.564)	0.049	1.173 (0.508–2.711)	0.709
Cause of defect				
Diabetes mellitus	1.000			
Trauma	0.651 (0.291–1.460)	0.298		
Others^1^	0.511 (0.233–1.120)	0.093		
Hemoglobin	0.751 (0.637–0.884)	0.001	0.882 (0.709–1.096)	0.257
White blood cell	1.104 (1.006–1.211)	0.036	1.082 (0.982–1.193)	0.112
Prognostic nutritional index^2^ (<40)	3.696 (1.869–7.307)	<0.001	3.859 (1.942–7.667)	<0.001
Duration of operation	1.002 (0.999–1.004)	0.199		
Crystalloid administered	1.000 (1.000–1.000)	0.072		
Colloid administered	1.000 (0.999–1.001)	0.943		
Flap perforator				
Skin	1.000			
Fasciocutaneous	7.147 (0.718–71.168)	0.094		
Musculocutaneous	5.835 (0.788–43.221)	0.081		

^1^Tumors, chronic osteomyelitis, scar adhesion, and arteriovenous malformation. ^2^Calculated as follows: 10 × serum albumin (g/dL) + 0.005 × total lymphocyte count (per mm^3^). Abbreviations: CI, confidence interval; ASA, American Society of Anesthesiologists.

**Table 4:** Incidence and odds ratio of free flap failure as a function of sex and a low PNI.

	Total Patients	Incidence of Free Flap Failure	Odds Ratio (95% CI)	*p*-Value
Men without a low PNI	264	5	1	
Women without a low PNI	151	9	3.283 (1.080–9.984)	0.036
Men with a low PNI	142	14	5.666 (1.997–16.075)	0.001
Women with a low PNI	68	10	8.931 (2.941–27.117)	<0.001

Data are presented as number. The PNI was calculated as 10 × serum albumin (g/dL) + 0.005 × total lymphocyte count (per mm^3^), with a PNI <40 defined as low. Abbreviations: CI, confidence interval; PNI, prognostic nutritional index.

**Table 5:** Postoperative outcomes.

Variables	Free Flap Failure (*n* = 38)	Free flap Survival (*n* = 587)	*p-*Value
Length of hospital admission (days)	62.1 ± 55.5	28.3 ± 24.4	<0.001
Rate of ICU admission	5 (13.2)	49 (8.3)	0.306
Length of ICU admission (days)	0.13 ± 0.34	0.08 ± 0.28	0.307

Continuous variables are presented as mean ± standard deviation and categorical variables as number (percent). Abbreviation: ICU, intensive care unit.
